# Epithelial Cell Adhesion Molecule in Primary Sjögren's Syndrome Patients: Characterization and Evaluation of a Potential Biomarker

**DOI:** 10.1155/2019/3269475

**Published:** 2019-12-05

**Authors:** Kui Zhang, Yaxin Zhou, Xiaojing Cheng, Xianghui Fu, Wanglei Du, Yuan Feng, Junfeng Jia, Xichao Yang, Guangzhi Xiao, Zhaohui Zheng, Ping Zhu, Zhenbiao Wu

**Affiliations:** ^1^Department of Clinical Immunology, Xijing Hospital, Air Force Medical University (Fourth Military Medical University), China; ^2^Department of Rheumatology, The Second Affiliated Hospital of Xi'an Jiaotong University, China

## Abstract

**Objective:**

To determine the subcellular localization of epithelial cell adhesion molecule (EpCAM) in labial salivary gland (LSG) and evaluate the diagnostic use of the extracellular domain of EpCAM (EpEX) and intracellular domain (EpICD) for primary Sjögren's syndrome (pSS).

**Methods:**

Immunohistochemical (IHC) analysis was conducted using EpEX and EpICD domain-specific antibodies on labial salivary gland biopsy (LSGB) from participants. Chi-square or Fisher's exact analysis, Mann–Whitney *U*-test, and Kruskal-Wallis test compared differences among groups. Independent risk factors of pSS were determined by multiple logistic regression analysis. Receiver-operator characteristic curves (ROC) were carried out to estimate the diagnostic value.

**Results:**

Compared to non-SS controls, loss of membranous EpEX and EpICD expression was observed in LSGB of pSS patients, which occurred in parallel with increased accumulation of cytoplastic and nuclear EpICD. The subcellular EpEX/EpICD expressions were associated with various features of pSS patients, especially histopathological grade of LSGB. Furthermore, high IHC scores of membranous EpEX were independent risk factors for pSS, even for the pSS patients at early stage. The IHC scores of subcellular EpEX/EpICD were of great diagnostic value for pSS with high sensitivity (70-80%) and specificity (85-95%).

**Conclusion:**

This study first found the aberrant expression pattern of EpCAM in LSG of pSS patients. The IHC scores of subcellular EpEX/EpICD were demonstrated to have the potential to act as diagnostic biomarkers for pSS.

## 1. Introduction

Primary Sjögren's syndrome (pSS) is a chronic inflammatory autoimmune disease characterized by focal lymphocytic sialadenitis (FLS) of exocrine gland [1]. Lymphocytes infiltrating and loss of tissue architecture are usually observed in labial salivary gland biopsy (LSGB) of patients, and focus score (FS) ≥ 1 relating to pSS is widely accepted in classification criteria [2–4]. However, LSGB usually identify pSS at more advanced stages of disease when gland damage has already occurred [4]. Though there are many other laboratory tests to help diagnose pSS, the fact is that diagnosis remains difficult for clinical practice, which always leads to delayed diagnosis and treatment [5]. The incidence of pSS is reported to be 9.92 per 100 000 people each year around the world [6], and the incidence rate of pSS in China is 0.33%-0.77% [7]. Delayed diagnosis not only causes poor prognosis for pSS patients but also aggravates the socioeconomic burden. Therefore, it emphasizes the essentiality to improve the diagnostic tools for pSS [8].

Considering early diagnosis for pSS is of great importance to prevent or decrease the occurrence of systematic complications [9–11], it is an urgent need to find a novel method or biomarkers to facilitate earlier diagnosis of pSS [5, 12]. In fact, the organ-specific biomarkers for pSS such as antisalivary gland protein 1 (SP1), anticarbonic anhydrase 6 (CA6), and antiparotid secretory protein (PSP) have been demonstrated to identify those patients at early disease, who were detected negative anti-Ro/SSA or anti-La/SSB [13]. However, there have been few researches on histopathological biomarkers directly correlating to the pathological development of labial salivary gland (LSG).

Epithelial cell adhesion molecule (EpCAM) is a glycosylated, 30 to 40 kDa type I membrane protein, comprising an extracellular domain (EpEX), a single transmembrane domain, and a short intracellular domain called EpICD [14]. In fact, EpCAM has been found to be expressed in most types of epithelia, engaging in cell proliferation, migration, invasion, cell cycle regulation, and cancer initiation [15–21]. Based on the multifunction of EpCAM engaged in epithelia, the researches into EpEX and EpICD have been widely reported in various epithelial diseases such as thyroid, prostate, colon, and oral cancer, which emphasized the strong correlation between EpCAM and epithelial diseases [22–26]. However, the expression pattern of EpCAM in LSG of pSS patients is still unknown.

Notably, a series of researches has suggested that EpCAM can be used as diagnostic or prognostic biomarkers for various cancers [22, 27, 28]. We thus proposed whether it could act as histopathological biomarkers for pSS. In the present study, we aimed to analyze the relationship between subcellular EpEX/EpICD and characteristics of pSS patients and further evaluate its diagnostic potential for pSS.

## 2. Materials and Methods

### 2.1. Participants

67 participants from Xijing Hospital during March 2013 to May 2016 were enrolled in the study. Our study was approved by the Ethics Committee of the Xijing Hospital, and informed consent was obtained from all participants. The ethics approval number is KY-20163016-1. The patients who have LSGB document were included, and all of those patients have complained of dry eyes and/or dry mouth or have been detected positive autoantibodies. The patients were excluded when they have (1) past head and neck radiation treatment, (2) sarcoidosis, (3) amyloidosis, (4) preexisting lymphoma, (5) hepatitis C infection, (6) acquired immunodeficiency disease (AIDS), (7) graft-versus-host disease, (8) used anticholinergic drugs (since a time shorter than 4-fold the half-life of the drug), and (9) IgG4-related disease.

Diagnosis was based on the 2002 American-European Consensus Group (AECG) classification criteria for pSS [2]; 47 patients were diagnosed as pSS while 20 were not. The degree of histopathological stage was graded from 0 to 4 according to Chisholm and Mason's standards [29]. The participants were divided into three groups according to their LSGB findings: histopathological grade 0 was found in 20 non-SS patients, grade 1 or 2 was found in 16 pSS patients, and grade 3 or 4 was found in 31 pSS patients. As previously described, the pSS patients were regarded as being in the early and advanced stages of SS, respectively [30].

### 2.2. Clinical Data and Collection of LSG

Apart from the diagnostic indicators (LSGB, anti-SSA, anti-SSB, ANA, and RF) mentioned in AECG or ACR [2, 3], we chose age, gender, duration of disease, anti-Ro52, IgG, and erythrocyte sedimentation rate (ESR) into the retrospective clinical data study, which were once reported to be associated with pSS [1, 31–35]. LSG tissue samples were obtained with informed consent, from individuals who underwent LSGB during their diagnostic evaluation for pSS.

### 2.3. Assessment of LSGB

The doctors obtained LSGBs following the method as previously described [36]. The method to obtain LSGBs and assess FS was referred to standardized consensus guidance [37]. The specimens were fixed in formalin, embedded in paraffin, and cut serially, divided into three groups for hematoxylin and eosin (HE), EpEX, and EpICD staining. Two experienced pathologists would first confirm the FLS of LSGBs and then assess FS. Finally, they evaluated the histopathological degree of LSGBs from 0 to 4 based on FS according to Chisholm and Mason's standards [29].

### 2.4. Immunohistochemical Staining

The sections (3 *μ*m thickness) of LSG tissues were deparaffinized and hydrated in xylene and graded alcohol series. Antigen retrieval was carried out with bath heating in 0.01 M citrate buffer, pH 6.8; endogenous peroxidase activity was blocked by incubating sections in methanol containing 0.3% hydrogen peroxide for 20 minutes. After blocking for nonspecific binding with mouse or rabbit serum, the sections were incubated with mouse polyclonal IgG anti-EpEX (dilution 1 : 10000, Department of Immunology, the Fourth Military Medical University, China) [38] and rabbit polyclonal IgG anti-EpICD (dilution 1 : 3000, Abcam, Cambridge, UK; cat no. ab71916). Staining was performed through the labeled streptavidin-biotin method (Histostain™-Plus Kits). Diaminobenzidine was used as the chromogen. Hematoxylin was used as the counterstain for nuclei [39].

### 2.5. Assessment of IHC Score

The IHC score of EpEX and EpICD was evaluated in the five most pathologically aggressive areas in the high-power field of the LSG tissue sections; for each field, we evaluated about 200~400 epithelial cells. EpEX and EpICD were evaluated in the acinar cell membrane, cytoplasm, and nucleus, respectively, based on the staining intensity and percentage of positive cells. The evaluation was independently carried out by two experienced pathologists in a blinded manner. For membranous EpEX and EpICD, the positive cells were defined as not full-circle-stained cells. For the cytoplasmic and nuclear EpICD, positive cells indicated the cells which showed staining in plasma or nucleus. These sections were scored as follows: 0, <10% cells; 1, 10–30% cells; 2, 31–50% cells; 3, 51–70% cells; and 4, >70% cells. And the intensity of membranous EpEX/EpICD staining was scored as follows: 0 = intense, 1 = moderate, 2 = mild, and 3 = none. For cytoplasmic and nuclear EpICD staining analysis, sections were scored on the basis of intensity as follows: 0 = none, 1 = mild, 2 = moderate, and 3 = intense. A final IHC score (ranging from 0 to 7) was the average of adding the scores of percentage and intensity of the five sections.

### 2.6. Statistical Analysis

For the variables documented in clinical data of participants, continuous data were expressed as median and extremes while frequencies as numbers and percentages by Microsoft excel. We used SPSS 21.0 software and GraphPad Prism 5.0 software to analyze statics. Chi-square or Fisher's exact analysis, Mann–Whitney *U*-test, and Kruskal-Wallis test were carried out to compare differences. Multiple logistic regression analysis was conducted to find the independent risk factors of pSS. *P* value < 0.05 was considered significant for statistical analysis. The cutoffs were based on the optimal sensitivity and specificity through receiver operating curve (ROC) analysis.

## 3. Result

### 3.1. Demographic, Laboratory, and Clinical Characteristics of Participants

**Figure 1:**
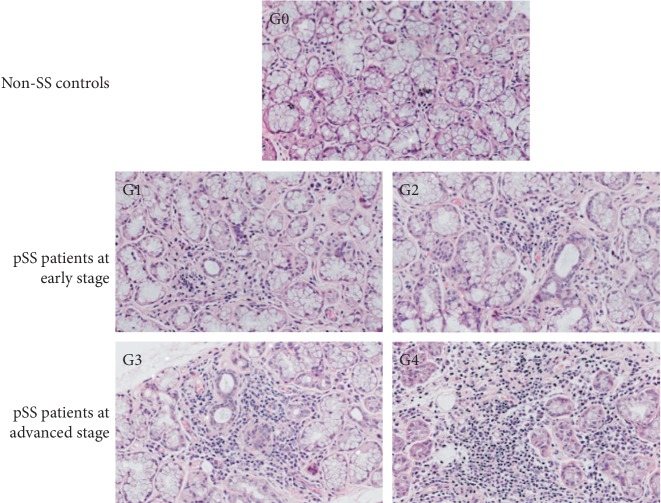
Pathology of labial salivary gland from the participants. The degree of histopathological stage was graded from 0 to 4 according to Chisholm and Mason's standards. Absent lymphocytes were observed in the LSGB at G0 from non-SS controls. In the LSGB of the pSS patients at early stage, slight or moderate infiltrate but less than one focus was observed, which was graded as G1 or G2. And the LSGB from the pSS patients at advanced stage showed one focus or more than one focus was graded as G3 or G4.

Table 1 exhibits the demographic, laboratory, and clinical characteristics of the participants in this study. The participants were divided into three groups by their histopathological grades, which were defined based on the lymphocytes per 4 mm^2^ [29].  Figure 1 exhibits the LSGB results of non-SS controls, pSS patients at early stage, and pSS patients at advanced stage.

In terms of demographic characteristic, all the subsets of pSS patients showed higher percentage of female sex but not the age at inclusion compared to non-SS controls. Additionally, the pSS patients at advanced stage suffered longer disease duration than the controls. As for the typical symptoms of pSS, all the subsets of pSS patients have complained more frequently about saprodontia but not xerophthalmia than the non-SS controls. And the prevalence of xerostomia in pSS patients except for who in the early disease was higher. As for autoantibodies, the pSS patients at early stage only showed higher positivity of anti-SSA, while the pSS patients at advanced or whole stage had higher positivity of anti-SSA, ANA, and anti-Ro52. More frequent detection of positive RF was only in the pSS patients at advanced stage. However, the positive anti-SSB detection in neither of the subsets of pSS patients has differed with the controls. Besides, all of the pSS patient subsets did not show significantly higher levels of IgG or ESR.

### 3.2. Aberrant Expression Pattern of EpCAM Was Detected in the LSG of pSS Patients

**Figure 2:**
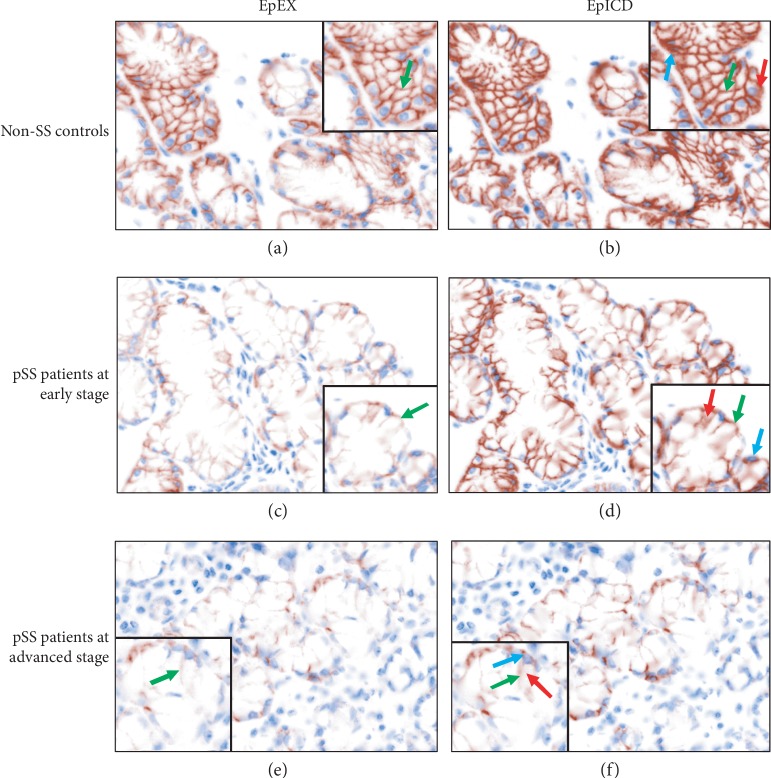
Immunochemical staining of EpEX and EpICD of labial salivary gland from the participants. The green arrow points membranous staining, the red arrow points cytoplasmic staining, and the blue arrow points nuclear staining.

The IHC staining of EpEX or EpICD on LSGB is shown in Figures 2(a)–2(f). In the normal tissue (Figures 2(a) and 2(b)), EpEX and EpICD could be observed in the membrane of LSG acinar cells. And EpICD could be also detected in the cytoplasm and nucleus. Compared with the IHC staining in the LSGB at G0, reduced membranous staining of both EpEX and EpICD was found in the acinar cells from the pSS patients at early disease with LSG at G1-2; meanwhile, the cytoplasmic and nuclear EpICD were more intense (Figures 2(c) and 2(d)). In the LSG cells from the pSS patients at advanced stage, mild staining of membranous EpEX and EpICD occurred while more frequent cytoplasmic and nuclear EpICD was observed (Figures 2(e) and 2(f)).

**Figure 3:**
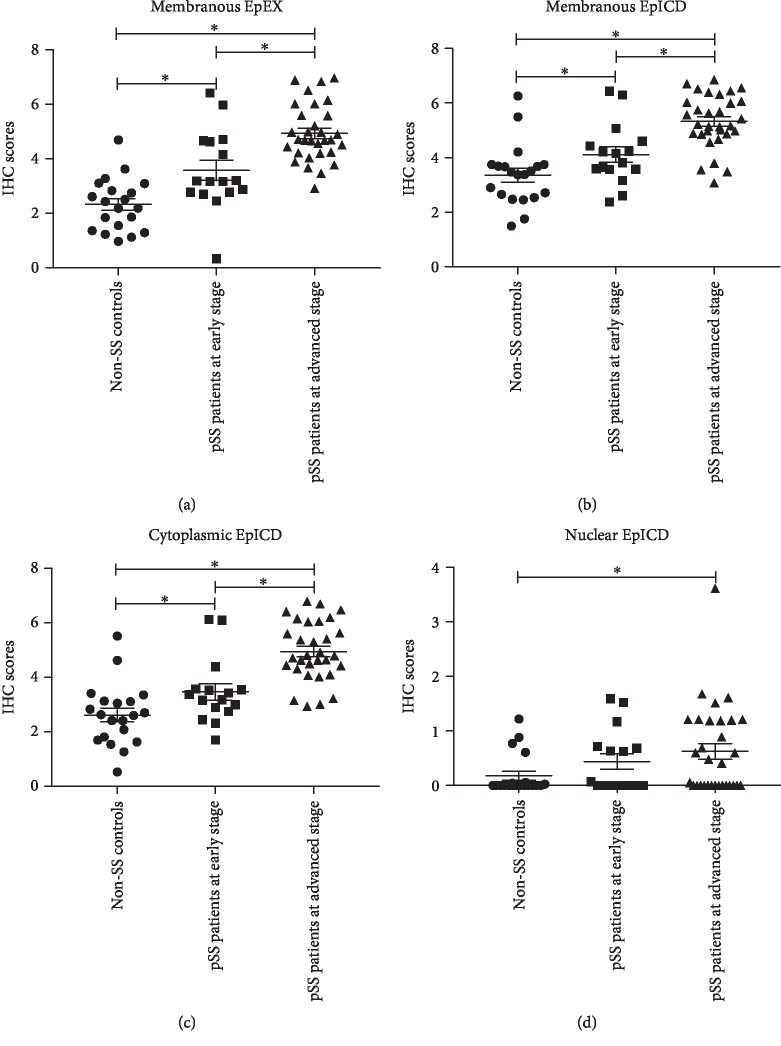
IHC scores of subcellular EpEX and EpICD in the labial salivary gland cells of non-SS controls, pSS patients at early stage, and pSS patients at advanced stage. (a) IHC scores of membranous EpEX among participants; (b) IHC scores of membranous EpICD among participants; (c) IHC scores of cytoplasmic EpICD among participants; (d) IHC scores of nuclear EpICD among participants. ^∗^The IHC scores were significantly different between groups with *P* < 0.05.

Figures 3(a)–3(d) show the IHC scores of EpEX or EpICD in the LSG of the participants. The mean ± SEM IHC scores of membranous EpEX of LSG tissues were 2.32 ± 0.96, 3.56 ± 1.47, and 4.93 ± 1.04 in the non-pSS controls, pSS patients at early stage, and pSS patients at advanced stage accordingly The mean ± SEM IHC scores of membranous EpICD were 3.36 ± 1.12, 4.11 ± 1.12, and 5.33 ± 0.97 while those of cytoplasmic EpICD were 2.62 ± 1.14, 3.47 ± 1.20, and 4.95 ± 1.08 among the LSG from the three groups of participants. And the IHC scores of nuclear EpICD were 0.18 ± 0.36, 0.43 ± 0.57, and 0.62 ± 0.81, which showed a high dispersion degree. Additionally, the comparison analysis further demonstrated that the membranous EpEX or EpICD and cytoplasmic EpICD IHC scores were different among the three groups of participants. And the nuclear EpICD IHC scores were only different between non-pSS controls and pSS patients at advanced stage.

### 3.3. IHC Scores of Subcellular EpEX and EpICD Were Associated with pSS Patients of Specific Features

**Figure 4:**
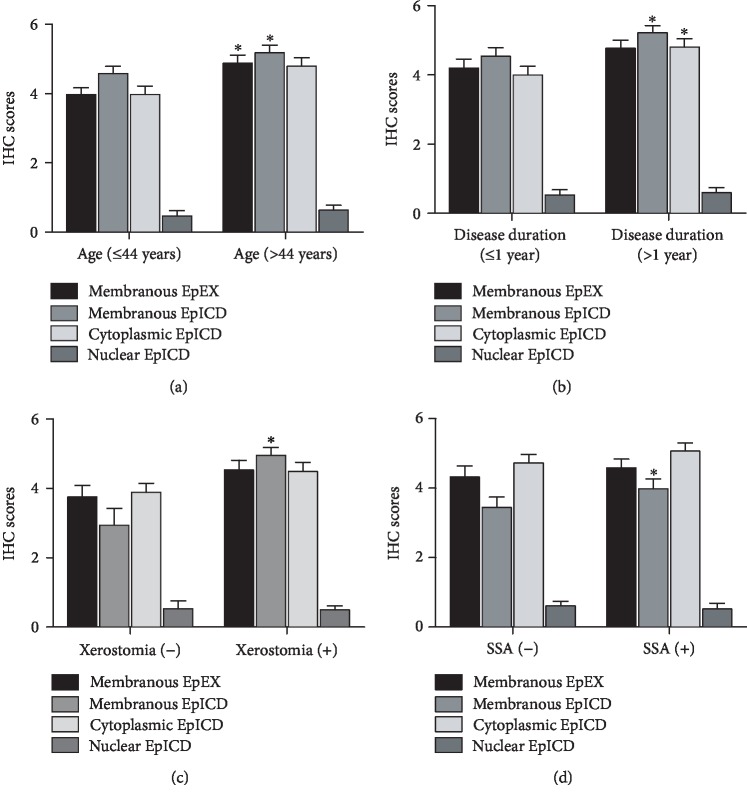
The associations between IHC scores of subcellular EpEX/EpICD and characteristics of pSS patients. (a) The association between IHC scores of subcellular EpEX/EpICD and age; (b) the association between IHC scores of subcellular EpEX/EpICD and disease duration; (c) the association between IHC scores of subcellular EpEX/EpICD and xerostomia; (d) the association between IHC scores of subcellular EpEX/EpICD and SSA antibody. ^∗^The IHC scores were significantly different between groups with *P* < 0.05.

To explore whether the expression of subcellular EpEX or EpICD was associated with typical features of pSS patients mentioned in Table 1, we thus conducted the comparison analysis of IHC scores of subcellular EpCAM among pSS patients with different features. Figures 4(a)–4(d) exhibit the features of pSS patients which were associated with EpEX or EpICD IHC scores.  Figure 4(a) showed that the IHC scores of membranous EpEX and EpICD tended to be higher in the pSS patients over 44 years old. And the patients who suffered the disease over 1 year usually had higher IHC scores of membranous and cytoplasmic EpICD (Figure 4(b)). Among the patients who complained of xerostomia, membranous IHC scores of EpICD in LSG were higher (Figure 4(c)). And in the anti-Ro/SSA positive patients, membranous IHC scores of EpICD were higher (Figure 4(d)).

### 3.4. High IHC Score of Membranous EpEX Was an Independent Risk Factor for pSS

The features of samples including gender, disease duration, xerostomia, saprodontia, anti-Ro/SSA, ANA, ANA and RF double positive, and anti-Ro52, which have been demonstrated to relate to pSS (Table 1), were enrolled in multiple logistic regression, together with IHC scores of subcellular EpCAM. As for pSS patients at early stage, xerostomia, ANA, ANA and RF double positive, anti-Ro52, and IHC score of nuclear EpICD were excluded from the regression analysis because they did not show significant difference between non-SS controls and pSS patients at early stage. The result shown in Table 2 indicated that high IHC score of membranous EpEX was an independent risk factor for pSS even for those patients at early stage. The OR value of membranous EpEX IHC score was 10.587 and 6.115 for pSS patients and the patients at early disease, respectively.

### 3.5. Subcellular EpEX and EpICD IHC Scores Have High Sensitivity and Specificity for Diagnosis of pSS

**Figure 5:**
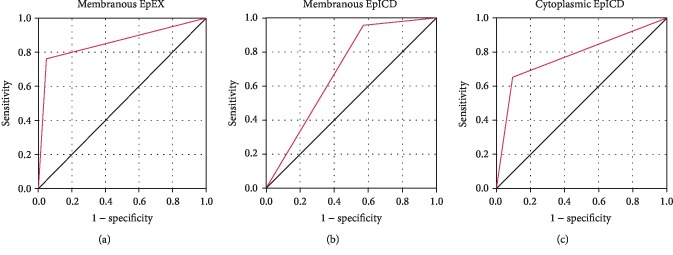
ROC analysis of IHC scores of subcellular EpEX/EpICD for pSS patients. (a) ROC analysis of membranous EpEX; (b) ROC analysis of membranous EpICD; (c) ROC analysis of cytoplasmic EpICD.

ROC curves shown in Figure 5 were generated for IHC scores of subcellular EpCAM in LSG tissues of pSS patients and controls. The result of nuclear EpICD was not shown because *P* > 0.05. Based on the analysis, the optimal cutoffs for the IHC scores of membranous EpEX, membranous EpICD, and cytoplasmic EpICD were determined as 3.640, 3.770, and 3.145, which could be used to distinguish the LSGB of SS patients from non-SS patients with high sensitivity and specificity. The biomarker analysis summarized in Table 3 showed that membranous EpEX could distinguish the pSS patients from controls with sensitivity of 74.47% and specificity of 95%. As for EpICD, the sensitivity of membranous EpICD was higher than cytoplasmic EpICD (78.72% vs. 72.34%) and the specificity was lower (85.00% vs. 95.00%). The AUC values were found to be 0.907 for membranous EpEX, 0.832 for membranous EpICD, and 0.864 for cytoplasmic EpICD.

## 4. Discussion

PSS is a chronic, progressive autoimmune disease that involves exocrine glands and results in dysfunctional impairment [31]. Delayed diagnosis always leads to the occurrence of systematic complications and poor prognosis for patients, which is the main concern of doctors [9–11]. Therefore, it is necessary to seek novel biomarkers to facilitate early diagnosis. Noticing that aberrant expression of EpCAM is a frequent event in epithelial diseases [39–41], we wondered if it also occurred in pSS. In this study, the expression pattern of EpCAM was found changed in the LSG acinar cells of pSS patients, and the IHC scores of membranous and cytoplasmic EpEX/EpICD had high sensitivity (70-80%) and specificity (85-95%) in diagnosis for pSS, supporting that IHC scores of subcellular EpCAM had a potential to act as diagnostic biomarkers, which would probably facilitate to make more accurate and earlier diagnosis.

It is well known that pSS overwhelmingly affects middle aged women, with a female to male ratio in incidence of approximately 9 : 1. The gender and age of pSS patients in our study coincided to the epidemiology reports [6]. Dry eyes (xerophthalmia), dry mouth (xerostomia), and saprodontia are the typical symptoms of pSS patients [42]. In our study, the pSS patients including those at advanced disease had more frequent complaints of xerostomia and saprodontia than non-pSS controls, which suggest that the symptoms of pSS is not obvious in early disease. In addition, the patients with other rheumatologic disease such as fibromyalgia could also complain of xerophthalmia or xerostomia [43]. Thus, we need more reliable objective indicators other than clinical signs to define the diagnosis of pSS.

In terms of serological profile, it is widely accepted that anti-Ro/SSA and anti-La/SSB are serum hallmarks for pSS [3]. The positivity of anti-Ro/SSA and anti-La/SSB in the total pSS patients (57.4%, 27.7%) of our study was consistent with previous studies [44]. In the 2016 ACR-EULAR Classification Criteria for pSS, anti-Ro/SSA was the only autoantibody included in the criteria, which weighted equally to LSGB result. In this study, anti-Ro/SSA was also the only serological indicator that all of the three subsets of patients have showed higher positivity than controls. A recent report has suggested that anti-Ro/SSA correlated with longer disease duration and higher intensity of lymphocytic infiltrates invading the LSG [44], while the SSB-positive/SSA-negative antibody profile is not associated with key phenotypic features of SS [45]. Similar results were also found in our study that the pSS patients with more severe pathology of LSG had a higher positivity of anti-Ro/SSA (*P* = 0.014) and longer disease duration (*P* = 0.02). The positivity of both ANA and RF was considered to indicate the diagnosis of pSS [3], and anti-Ro52 autoantibody testing may help to identify a specific subset of SS patients with more aggressive disease [46]. In this study, the more severe pSS patients showed higher prevalence of ANA, ANA and RF double positive, and anti-Ro52. In fact, the levels of ESR and IgG in pSS patients were frequently higher than healthy people [35], which did not reach significantly higher in our study. The results probably on account of that the 20 non-pSS controls may have other rheumatologic or inflammatory diseases that caused the ESR and IgG levels increased. It needs to be clarified that the patients with other autoimmune diseases such as rheumatoid arthritis, which may show SSA/SSB, ANA, or RF positive, have been excluded in the patient group.

The results of comparison analysis above suggested that the pSS patients at early stage were hard to recognize because the symptoms of whom were not obvious and the positivity of autoantibodies was low, which emphasized the importance to explore novel biomarkers directly relating to pSS. EpCAM, a 40 kDa transmembrane glycoprotein, is one of the most widely investigated proteins in human [14]. In this study, we found decreased membranous accumulation of EpEX and EpICD, while increased location of cytoplasmic and nuclear EpICD in the LSG acinar cells infiltrated by lymphocytes of pSS patients. Similar findings of the EpCAM expressive changes have been reported in various types of epithelial diseases [23, 28, 39, 40], which suggest the aberrant expression pattern of EpCAM is a common event happened in the pathology of epithelium. Functionally, EpCAM is found to be activated by regulated intramembrane proteolysis (RIP) and then acts as a mitogenic signal transducer, which involves nuclear translocation of EpICD and then increases transcription of the target genes such as c-myc [47, 48], which could promote cell proliferation [49]. However, recent studies have revealed that c-myc is also an important apoptotic regulator [50]. It is well known that LSG cell apoptosis is one of the most important causes for the pathogenesis of pSS, which is tightly controlled by cytotoxic mediators and cell survival molecules [51]. Interestingly, c-myc mRNA expression has been found upregulated in the minor salivary gland of pSS patients [52]. Those investigations provide us new clues to explore whether the EpCAM signal pathway could induce the LSG cell apoptosis in the patients with pSS.

We used a semiquantitative method to evaluate the expression pattern of EpCAM. And the results of comparison analysis indicated that the IHC scores of subcellular EpCAM were different among the non-SS controls, pSS patients at early stage, and pSS patients at advanced stage. In fact, the IHC scores of subcellular EpEX or EpICD were not only related to the histopathological grade of LSG but also associated with some characteristics of pSS patients such as age, disease duration, xerostomia, and anti-Ro/SSA, which suggested the close connection between EpCAM and pSS. Furthermore, the membranous IHC score of EpEX in LSG acinar cells was further validated to be the independent risk factors for pSS patients (*P* < 0.01), even for the pSS patients at early stage (*P* < 0.05). Next, we proposed that IHC staining results of subcellular EpEX and EpICD have a great value to make the diagnosis of pSS easier and earlier. And the ROC analysis validated our hypothesis that the IHC scores of membranous both EpEX and EpICD and cytoplasmic EpICD had the potential to be used as diagnostic biomarkers for pSS with high sensitivity and specificity. Among them, the IHC score of membranous EpICD showed the highest sensitivity (78.72%) and membranous EpEX had the highest specificity (95.00%).

In conclusion, this study first revealed the aberrant expressive pattern in LSG acinar cells of patients with pSS, which closely associated with some characteristics of pSS. And the IHC scores of membranous EpEX was the independent risk factor for pSS patients including those at early stage. However, we have to admit that there are some deficiencies existing in our study such as lacking a larger cohort of pSS patients to validate our findings and assessment method of IHC scores requiring to be more objective and reliable. Taken together with the findings of our study, IHC scores of subcellular EpCAM had a potential to be biomarkers of pSS diagnosis with high sensitivity and specificity, which is of great importance for patients to get earlier diagnosis and better prognosis.

## Figures and Tables

**Table 1:** Demographic, clinical, and serological features of study participants.

	Non-SS controls	pSS patients at early stage	*P* value	pSS patients at advanced stage	*P* value	pSS patients included in study	*P* value	
Histopathologic grade	G0, 20 (100%)	G1-2, 16 (100%)	<0.001	G3-4, 31 (100%)	<0.001	G1-2, 16 (34.0%), G3-4, 31 (66.0%)	<0.001
Female sex	4 (20%)	14 (87.5%)	<0.001	29 (93.5%)	<0.001	43 (91.5%)	<0.001
Age at the inclusion (year)	44 (17, 58)	44 (19, 45)	ns	52 (25, 80)	ns	50 (19, 80)	ns
Disease duration (year)	<1 (<1, 20)	<1 (<1, 10)	ns	4 (<1, 30)	0.02	2 (<1, 30)	ns
Xerophthalmia	6 (30%)	7 (43.8%)	ns	16 (51.6%)	ns	23 (48.9%)	ns
Xerostomia	10 (50%)	10 (62.5%)	ns	28 (90.3%)	0.001	38 (80.9%)	0.01
Saprodontia	2 (10%)	8 (50%)	0.011	16 (51.6%)	0.002	24 (51.1%)	0.002
Anti-Ro/SSA	4 (20%)	10 (62.5)	0.016	17 (54.8%)	0.014	27 (57.4%)	0.005
Anti-La/SSB	6 (30%)	4 (25%)	ns	9 (29.0%)	ns	13 (27.7%)	ns
ANA	12 (60%)	12 (75%)	ns	29 (93.5%)	0.010	41 (87.2%)	0.029
RF (≥IU/mL)	7 (35%)	7 (43.8%)	ns	20 (64.5%)	0.039	27 (57.4%)	ns
ANA and RF	4 (20%)	5 (31.3%)	ns	20 (64.5%)	0.002	25 (53.2%)	0.012
Anti-Ro52	6 (30%)	9 (56.3%)	ns	18 (58.1%)	0.05	27 (57.4%)	0.04
IgG (mg/dL)	1515 (15, 3830)	1875 (796, 3100)	ns	1975 (154, 4400)	ns	1900 (154, 4400)	ns
ESR (mm/hr)	30 (4, 104)	48.5 (2, 120)	ns	40 (4, 140)	ns	45 (2, 140)	ns

**Table 2:** Risk factor analysis for pSS patients.

	pSS patients			pSS patients at early stage	
Factors	OR	*P* value	95% CI	OR	*P* value	95% CI
Membranous EpEX	10.587	**0.009**	1.797-62.382	6.115	**0.033**	1.154-32.414
Membranous EpICD	61.972	0.058	0.870-4416.473	0.139	0.287	0.004-5.260
Cytoplasmic EpICD	0.019	0.093	0.000-1.944	5.815	0.272	0.252-134.223
Nuclear EpICD	3.659	0.301	0.313-42.743			
Female	0.408	0.520	0.027-6.244	1.371	0.793	0.130-14.463
Xerostomia	0.469	0.589	0.030-7.335			
Saprodontia	5.354	0.171	0.485-59.043	9.028	0.058	0.928-87.849
Anti-SSA	0.784	0.684	0.277-0.222	0.761	0.522	0.330-1.754
ANA	0.437	0.337	0.022-5.236			
ANA and RF	1.304	0.837	0.104-16.394			
Anti-Ro52	1.195	0.867	0.150-9.531			

**Table 3:** Biomarker analysis of subcellular EpEX/EpICD IHC scores for pSS.

Non-SS controls (*n* = 20)			pSS patients (*n* = 47)		
IHC score	Cutoff	Sensitivity	Specificity	AUC	*P* value
Membranous EpEX	3.640	74.47%	95.00%	0.907	<0.001
Membranous EpICD	3.770	78.72%	85.00%	0.832	<0.001
Cytoplasmic EpICD	3.145	72.34%	90.00%	0.864	<0.001

## Data Availability

The experimental results which are used to support the findings of this study are included within the article. In order to verify our results and increase the reliability of our work, all the authors of this paper agree to share the data of this study. If the reviewers or editors require the original data, please contact immuZhouYaxin@outlook.com.

## Supplementary Materials

Supplementary Materials Table SI: histopathologic grade of the study participants. Table SII: risk factor analysis for pSS patients. Table SIII: risk factor analysis for pSS patients at early stage.
