# Supplementary material for: Epithelial Cell Adhesion Molecule in Primary Sjögren's Syndrome Patients: Characterization and Evaluation of a Potential Biomarker
Source: J Immunol Res. 2019 Dec 5;2019:3269475. doi: 10.1155/2019/3269475 (PMC6915146; doi:10.1155/2019/3269475)
Supplement: Supplementary Materials — Table SI: histopathologic grade of the study participants. Table SII: risk factor analysis for pSS patients. Table SIII: risk factor analysis for pSS patients at early stage. [file 3269475.f1.pdf]

Supplemental Table

Table SI. Histopathologic grade of the study participants.

	non-SS controls	pSS patients at early stage	P-value	pSS patients at advanced stage	P-value	pSS patients included in study	P-value
Histopathologic grade	G0,20(100%)<br>G1-2,0(0%)<br>G3-4,0(0%)	G0,0(0%)<br>G1-2,16(100%)<br>G3-4,0(0%)	<0.001	G0,0(0%)<br>G1-2,0(0%)<br>G3-4,31(100%)	<0.001	G0,0(0%)<br>G1-2,16(34.0%),<br>G3-4,31(66.0%)	<0.001

Table SII. Risk factors analysis for pSS patients.

	pSS patients		
**factors**	**OR**	**P-value**	**95%CI**
Membranous EpEX	10.587	**0.009**	1.797-62.382
Membranous EpICD	61.972	0.058	0.870-4416.473
Cytoplasmic EpICD	0.019	0.093	0.000-1.944
Nuclear EpICD	3.659	0.301	0.313-42.743
Female	0.408	0.520	0.027-6.244
Xerostomia	0.469	0.589	0.030-7.335
Saprodontia	5.354	0.171	0.485-59.043

Anti-SSA	0.784	0.684	0.277-0.222
ANA	0.437	0.337	0.022-5.236
ANA and RF	1.304	0.837	0.104-16.394
Anti-Ro52	1.195	0.867	0.150-9.531

Table SIII. Risk factors analysis for pSS patients at early stage.

	**pSS patients at early stage**		
**factors**	**OR**	**P-value**	**95%CI**
Membranous EpEX	6.115	**0.033**	1.154-32.414
Membranous EpICD	0.139	0.287	0.004-5.260
Cytoplasmic EpICD	5.815	0.272	0.252-134.223
Female	1.371	0.793	0.130-14.463
Saprodontia	9.028	0.058	0.928-87.849
Anti-SSA	0.761	0.522	0.330-1.754
